# Distinct neural mechanisms construct classical versus extraclassical inhibitory surrounds in an inhibitory nucleus in the midbrain attention network

**DOI:** 10.1038/s41467-023-39073-5

**Published:** 2023-06-09

**Authors:** Hannah M. Schryver, Shreesh P. Mysore

**Affiliations:** 1 Department of Psychological and Brain Sciences, Johns Hopkins University, Baltimore, MD 21218 USA; 2 Currently, Allen Institute, Seattle, WA USA; 3 The Solomon H. Snyder Department of Neuroscience, Johns Hopkins School of Medicine, Baltimore, MD 21205 USA; 4 Kavli Neuroscience Discovery Institute, Johns Hopkins University, Baltimore, MD 21218 USA

**Keywords:** Neural circuits, Sensory processing, Visual system

## Abstract

Inhibitory neurons in the midbrain spatial attention network, called isthmi pars magnocellularis (Imc), control stimulus selection by the sensorimotor and attentional hub, the optic tectum (OT). Here, we investigate in the barn owl how classical as well as extraclassical (global) inhibitory surrounds of Imc receptive fields (RFs), fundamental units of Imc computational function, are constructed. We find that focal, reversible blockade of GABAergic input onto Imc neurons disconnects their extraclassical inhibitory surrounds, but leaves intact their classical inhibitory surrounds. Subsequently, with paired recordings and iontophoresis, first at spatially aligned site-pairs in Imc and OT, and then, at mutually distant site-pairs within Imc, we demonstrate that classical inhibitory surrounds of Imc RFs are inherited from OT, but their extraclassical inhibitory surrounds are constructed within Imc. These results reveal key design principles of the midbrain spatial attention circuit and highlight the critical importance of competitive interactions within Imc for its operation.

## Abstract (web summary)

Imc is a midbrain inhibitory nucleus essential for stimulus selection across space. Here, the authors show in the barn owl that global inhibitory surrounds of Imc neurons are constructed within Imc, but their classical inhibitory surrounds are inherited from inputs.

## Introduction

Animals behave in complex environments and are constantly faced with multiple competing stimuli. Selecting the location with the most “important” or highest priority stimulus to guide behavior at any instant is an essential part of adaptive behavior, and operates upon the foundation of a spatial map of relative stimulus priority^1–4^. Equally essential is the processing and representation of the stimulus at the selected location. For neurons involved in spatial selection, a core characteristic that impacts both these functions is their spatial receptive field (RF), defined as the subset of the spatial locations that a neuron responds to selectively. The excitatory center and classical inhibitory surround of the RF together control the responses of neurons to a stimulus inside the RF, whereas the extraclassical surround controls the modulation of the neuron’s responses by a competing stimulus outside the RF. Thus, understanding how classical and extraclassical surrounds are constructed is essential for understanding how neurons involved in selection achieve both competitive selection among multiple competing stimuli as well as the processing and representation of the selected target.

**Fig. 1:**
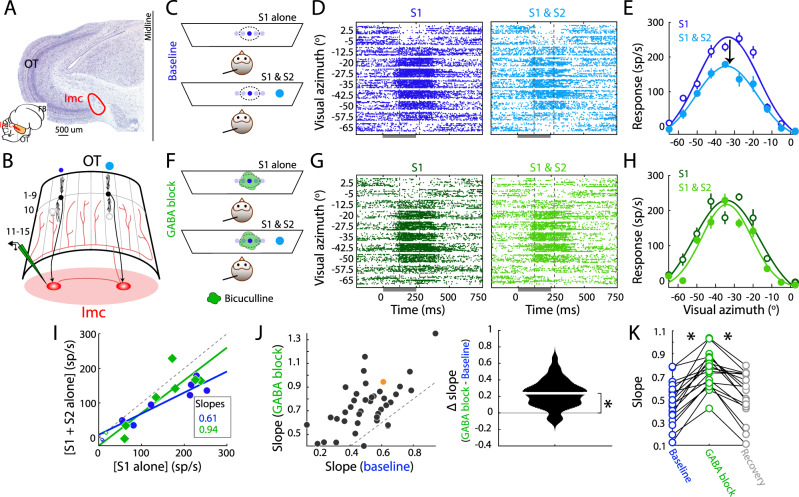
Imc’s extraclassical inhibitory surrounds are computed locally in Imc. **A** Inset: Schematic of barn owl brain. OT: optic tectum, Imc: nucleus isthmi pars magnocellularis, FB: forebrain. Vertical line: Coronal section. Main: Nissl-stained coronal section. “C”-shaped staining: OT layers. **B** Schematic of Imc-OT connectivity. Curved sheet: OT; numbers → layers (1–15). Columns: topographic encoding of adjacent azimuthal locations^14^. Blue dots: stimuli (S1 and S2) at distant azimuths (>30° apart)^20, 27^. Black neurons: OT layer 10 (OT_10_) neurons encoding these stimuli and providing excitatory input to Imc neurons (red ovals). Red lines: Inhibitory projections from Imc neuron on the right to OT intermediate/deep layers (11 to 15; OT_id_); projections target OTid space map broadly sparing only the portion of OT (here, columns) providing input^14, 32^. Horizontal red line: inhibition among Imc neurons^26, 33^. Projections of left Imc neuron not shown for clarity. Recording symbol: glass electrode for bicuculline methiodide (green) iontophoresis and recording. **C**–**E** Baseline condition: Measuring extraclassical (competitive) inhibitory surrounds of Imc neurons. **C** Stimulus protocol showing owls viewing a monitor, electrode in Imc (line), RF of Imc site (dotted oval). Dots: looming stimuli; S1- dark blue, near RF; S2-light blue, distant competitor; dot size: speed of loom (Methods). **D** Raster responses of example Imc site; dark gray bar: stimulus duration. RF center: (−31.7° azimuth, −7° elevation); S1 loom speed = 6°/s. S2 location: (+7° azimuth, 17° elevation); S2 loom speed = 10°/s. Negative azimuths => locations in left hemifield, contralateral to this recording site. Dashed vertical lines: window for firing rate estimation (125–275 ms). **E** Response firing rates (mean ± s.e.m). Solid lines: Gaussian fits. **F**–**H** GABA blockade condition: Same as (**C**–**E**), but during focal bicuculline iontophoresis (**F**, green blob) in Imc (Methods). **G**, **H** Responses of Imc site in D,E during GABA blockade. **I** Scatter plot of responses of the example Imc site to S1 versus to S1 and S2. Blue: baseline (from **E**). Green: GABA blockade (from **H**). Straight lines: Best linear fits to responses at locations within site’s RF (large, filled symbols). Slopes: 0.61 (baseline), 0.94 (GABA blockade), *p* = 0.044, one-sided permutation test; smaller values => greater competitive suppression^20, 27^. **J** Population summary. Left: GABA blockade vs. baseline slopes; *n* = 40 sites (after removing 4 outliers; Methods); S2 location = 33.8° ± 1.03° away from Imc RF center. Orange dot: example site in (**D**–**I**). Right: Violin plot of difference in slopes; median =0.24 (white line). ‘*’: *p* = 8 ×10^−8^, two-sided sign-rank test. **K** Recovery (summary). Slopes at a subset of Imc sites (*n* = 18) measured in baseline, GABA blockade, and recovery (bicuculline-off) conditions. ‘*’: significant, see text for test and *p*-values. See also Fig. S1. Source data are provided as a Source Data file.

The optic tectum (OT, or superior colliculus, SC, in mammals) is a major sensorimotor hub in the midbrain (Fig. 1A). SC/OT neurons encode space topographically, and are also known to encode a spatial map of relative stimulus priority^2,5–7^. The SC/OT is required for the control of spatial selection and selective attention when a target is present amidst distracters^8–10^, with OT neurons signaling the highest priority stimulus among competing stimuli categorically^11–13^. Notably, these competitive interactions within the OT are controlled by long-range inhibition generated by GABAergic neurons in the nearby isthmi pars magnocellularis (Imc; Fig. 1A, B)^14^. Specifically, focal inactivation of Imc neurons abolishes stimulus competition within the OT^15,16^. Additionally, recent results demonstrate that the signaling of the strongest stimulus by the Imc occurs earlier, and is more categorical than in the OT, further highlighting the importance of Imc to the function of the midbrain selection network.

In turn, Imc neurons, which have well-defined spatial receptive fields^17,18^, exhibit both classical inhibitory surrounds and extraclassical competitive surrounds^18–20^ Specifically, responses of Imc neurons to a bar stimulus of increasing length (or a circular stimulus of increasing size) have been shown to drop to low values at large lengths (sizes), demonstrating the presence of classical inhibitory surrounds^18^. In parallel, responses of Imc neurons to a stimulus inside the RF are divisively suppressed by a second stimulus anywhere outside the RF, demonstrating a global, extraclassical inhibitory surround. Whereas the source of the excitatory drive for Imc receptive fields is known to be neurons in layer 10 of the OT (OT_10_)^21^, how the inhibitory surrounds of Imc receptive fields are constructed is not understood. Addressing this question is key to understanding how the map of relative stimulus priority is constructed and stimulus selection orchestrated in the OT.

Here, we systematically dissect the mechanisms by which the inhibitory surrounds of Imc neurons in the barn owl are constructed. We do so in a series of experiments involving extracellular recordings in the Imc coupled with iontophoretic silencing of GABAergic input onto Imc neurons, silencing of GABAergic input onto spatially aligned OT sites, or silencing of excitatory (glutamatergic) input onto other/distant Imc sites. First, with iontophoretic blockade of GABAergic input on Imc neurons, we show that whereas global competitive surrounds of Imc neurons are abolished, the classical inhibitory surrounds are not affected. Then, with paired recording experiments at spatially aligned sites in the Imc and OT, we demonstrate that classical inhibitory surrounds of Imc RFs are controlled by inhibition onto OT_10_ neurons – the sole source of excitatory input to Imc neurons. Finally, with paired recording experiments at spatially misaligned sites within Imc, we demonstrate that global competitive surrounds of Imc RFs are controlled by long-range inhibition from distant Imc neurons. Our results reveal that distinct mechanisms are involved in the construction of the inhibitory surrounds of Imc neurons: classical, local inhibitory surrounds are conferred onto Imc neurons by OT_10_ neurons, while global, competitive surrounds are constructed within the Imc using inhibition from distant Imc neurons.

## Results

### Imc’s extraclassical inhibitory surrounds are computed locally in the Imc

We first investigated mechanisms underlying the global, extraclassical inhibitory surrounds exhibited by Imc neurons. Specifically, we asked if the reduction of stimulus-evoked Imc responses by a distant competitor presented outside the RF^19,20^ was due to a comparison occurring at the Imc site itself, or if this response reduction reflected computations occurring elsewhere.

To this end, we conducted extracellular recordings in the Imc using multibarrel glass iontophoresis electrodes filled with a bicuculline methiodide solution (Fig. 1B, Methods). We recorded tuning curve responses at Imc sites using a single stimulus (S1), or while simultaneously also presenting a second, stronger stimulus far outside a site’s RF (S2; >30° away; Fig. 1C). Both S1 and S2 were visual looming stimuli whose strengths are controlled by their loom speeds (Mysore et al., 2010; Methods; S1 strength: 6°/s, S2 strength 10°/s). Trials with S1 alone, or S1 and S2 presented simultaneously, were interleaved randomly. Consistent with previous findings, responses to paired S1 and S2 presentations were significantly reduced compared to responses to S1 alone (Fig. 1D, E, I; slope=0.61). We then repeated these measurements following iontophoresis of bicuculline at the Imc recording site (Fig. 1F, Methods), thereby blocking GABAergic synaptic transmission onto the recorded Imc neurons. We found that this nearly abolished the reduction of responses due to the competitor S2, thereby disconnecting this Imc site’s extraclassical surround (Fig. 1G–I; slope=0.94; *p* = 0.044, permutation test (GABA blockade vs. baseline slopes)).

Across a population of tested Imc sites (*n* = 40, following the removal of four outliers; Methods), we found that bicuculline iontophoresis at Imc consistently weakened this competitor-dependent response reduction (Fig. 1J, K; *p* = 8 × 10^−8^, sign rank test). We verified that these results were specifically due to drug iontophoresis by measuring responses following recovery from bicuculline iontophoresis at a subset of Imc sites (*n* = 18; Methods). We found that competitor-dependent response reduction returned to strong, near-baseline levels (Fig. 1K; baseline vs inactivation, *p* = 0.0002; inactivation vs recovery, *p* = 0.0002; baseline vs recovery, *p* = 0.064; sign rank tests followed by Holm-Bonferroni correction form multiple comparisons). This recovery occurred despite a small, but progressive reduction in the maximum evoked firing rates to S1 alone over time—from the baseline through recovery conditions, consistent with typical time-dependent run-down effects during extended electrophysiological recordings (Fig. S1A–D; Methods).

Together, these results demonstrated that the reduction of Imc responses by a distant competing stimulus is due to suppression caused by GABAergic synapses on these Imc neurons. Global competitive (inhibitory) surrounds are thus constructed locally at the Imc neurons themselves.

### Imc’s classical inhibitory surrounds are not computed locally in the Imc

**Fig. 2:**
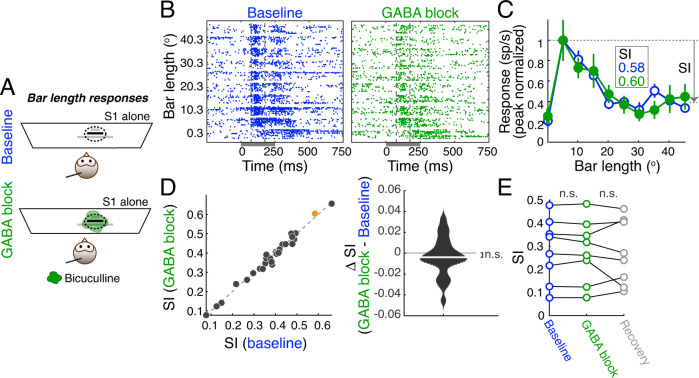
Imc’s classical inhibitory surrounds are not computed locally in the Imc. **A** Bar length protocol for assessing the effect of GABA blockade on Imc classical inhibitory surrounds: a bar stimulus of increasing length was presented inside the spatial RF of the Imc site (dashed oval), in the baseline (Top) and GABA blockade (Bottom) conditions. Bars were horizontal, centered at the azimuthal center of the RF, and had a fixed height of 3°. **B** Raster plots of responses of an example Imc site to bars of increasing lengths in the baseline (Left) and GABA blockade (Right) conditions. RF center of site: (7.53° azimuth, −10° elevation). Positive values of azimuth indicate contralateral locations for this site. Dark gray bar: Stimulus duration (250 ms). Dashed vertical lines: window of responses used for estimating response firing rates (75–175 ms). Other conventions as in Fig. 1D. **C** Firing rate responses (mean ± s.e.m) as a function of bar length (normalized to peak response), for site in (**B**). Blue (open): baseline; green (filled): GABA blockade. Suppression Index (SI) = asymptotic response suppression/peak response (indicated). SI = 0.58 (baseline), and 0.60 (GABA blockade); p = 0.438, one-sided permutation test; Methods. **D** Population summary. Left: Scatter plot of SI at baseline versus GABA blockade conditions (*n* = 28; after removal of 3 outliers; Methods). Orange dot: example site in (**B**, **C**). Right: Violin plot of difference between SI values of Imc sites in GABA blockade vs. baseline conditions; median SI value = −0.0039 (white line); ‘n.s.’: not significant; *p* = 0.28, two-sided sign rank test. **E** Recovery (summary). SIs at a subset of Imc sites (*n* = 8), measured in the baseline, GABA blockade, and recovery conditions. ‘ns’: not statistically significant; two-sided sign rank test, *p* < 0.01, see text for *p*-values. See also Fig. S2. Source data are provided as a Source Data file.

We next investigated mechanisms underlying the classical surrounds exhibited by Imc neurons. Specifically, we tested if inhibition impinging onto the neurons, a common mechanism underlying the classical inhibitory surrounds of cortical as well as sub-cortical neurons, mediates the construction of classical surrounds in Imc as well. To this end, we characterized the classical inhibitory surround using a protocol used extensively in the literature, including in Imc—measurement of responses to bar stimuli of systematically increasing lengths (Methods; Fig. 2A)^18,22,23^. This protocol is well-established to produce response profiles as a function of bar length in which firing rates rise to a peak before decreasing to an asymptotic value (Fig. S2A)^24^. This shape of the response profile is the result of the traditional structure of the spatial receptive field—the combination of a strong but spatially narrow excitatory center with a weaker but broader inhibitory surround (Fig. S2B). At small lengths, the excitatory center is activated more than the inhibitory surround, and is activated progressively more as bar length increases, producing the rising phase of response profile. This is followed, at larger bar lengths, by the fixed activation of the spatially limited excitatory center together with an increasing activation of the larger inhibitory surround, producing the falling phase (‘dip’) of the response profile. Finally, at sufficiently large bar lengths (exceeding the spatial extent of the inhibitory surround), there is fixed activation of both the excitatory center and the surround, producing the asymptotic phase of the response profile (Fig. S2A, B). The shape of the bar length response profile, therefore, reflects the strength and spatial extent of the classical inhibitory surround. Specifically, the amount of suppression of the asymptotic response with respect to the peak response, quantified as the suppression index (SI) = (peak response—asymptotic response)/peak response, is a reliable metric of the classical inhibitory surround, with smaller SI values signaling weaker surrounds^24,25^.

We measured bar-length response profiles (and SI values; Methods) without and with bicuculline iontophoresis (Fig. 2A) at Imc sites. Within each condition, bars of different lengths were presented in a randomly interleaved manner. Should inhibitory influences onto Imc neurons be involved in creating the classical inhibitory surround, we would expect blockade of this inhibition to substantially alter the bar-length response profile—causing a much smaller dip in responses at medium to large bar lengths and producing a smaller SI value.

We found that the responses of Imc sites to the bar-length protocol exhibited the classic shape (Fig. 2B, C; blue data). However, following bicuculline iontophoresis onto Imc, we found no change in the shape of the bar length response profile, and specifically, no change in the SI (Fig. 2B, C; green data, peak-normalized responses plotted to better visualize any potential changes in shape and SI; SI = 0.58 (baseline, blue), 0.60 (GABA blockade, green), *p* = 0.438; permutation test).

Across a population of Imc sites at which we presented bars of different lengths, we found no systematic effect of bicuculline iontophoresis on SI (Fig. 2D, *p* = 0.28, sign rank test; *n* = 28 sites, following the removal of three outliers; orange dot: example site in B; Methods). Notably, nearly all these sites (*n* = 24/28) were the same as those at which we also obtained the tuning curve data for Fig. 1, with the bar-length response profiles obtained in a randomly interleaved manner with the tuning curve data. We, therefore, were able to rule out the possibility that the drug was ineffective at blocking GABA synapses on Imc neurons in this experiment. Separately, consistent with the results in Fig. S1, we found that the peak evoked responses to bar lengths exhibited a progressive reduction over time, from the baseline through recovery conditions (Fig. S2C–F). Nonetheless, this response run-down did not affect SI, as demonstrated by nearly unchanged SI values across the baseline, GABA blockade and recovery conditions, measured in a subset (*n* = 8) of the sites (Fig. 2E; baseline vs inactivation, *p* = 0.38; inactivation vs recovery, *p* = 0.95; baseline vs recovery, *p* = 0.64; sign rank test followed by Holm–Bonferroni correction for multiple comparisons).

Taken together, these results established that GABAergic inhibition onto Imc neurons does not participate in the construction of their classical inhibitory surrounds.

### Imc’s classical inhibitory surrounds are inherited from the OT

**Fig. 3:**
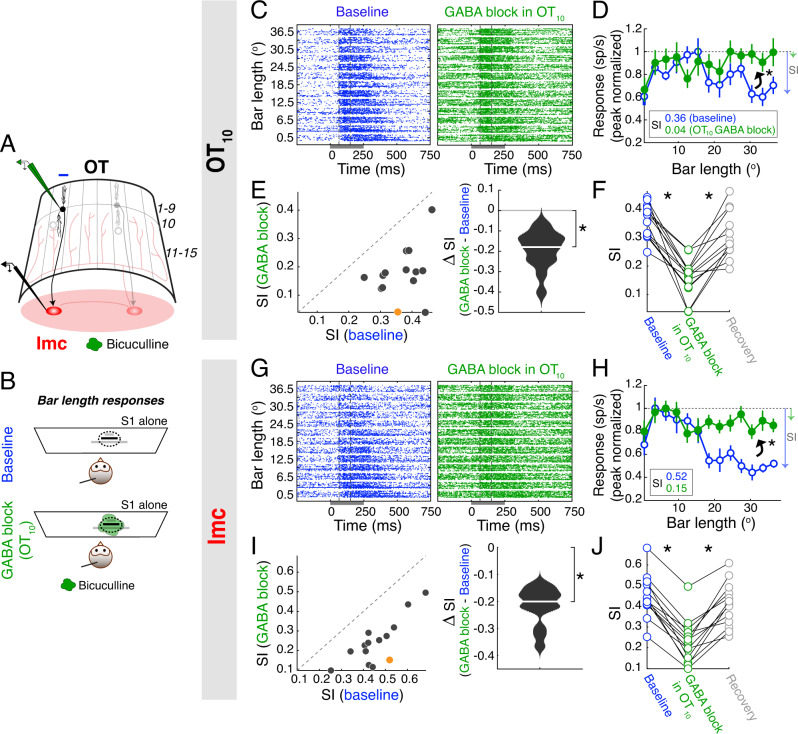
Imc’s classical inhibitory surrounds are inherited from OT_10_. **A** Imc-OT schematic showing glass iontophoretic electrode (green, bicuculline methiodide) in OT_10_, and a second, tungsten electrode (black) at a spatially aligned Imc site; other conventions as in Fig. 1B. **B** Stimulus protocol for measuring bar length response profiles (simultaneously in aligned Imc and OT_10_ sites) in the baseline condition (Top), and during GABA blockade in OT_10_ (Bottom); conventions as in Fig. 2A. **C**–**F** Bar length responses at OT_10_ sites. **C** Raster plots of responses of example OT_10_ site. RF center of site = (6.23° azimuth, 10° elevation); positive azimuth values indicate contralateral locations. Dashed vertical lines: 65–150 ms. Conventions as in Fig. 1D. **D** Firing rate responses of site in C (normalized to peak). Blue (open): baseline; green (filled): GABA blockade in OT_10_. SI = 0.36 (baseline), 0.04 (GABA blockade). ‘*’: *p* = 0.002, one-sided permutation test). Other conventions as in Fig. 2C. (E) Population summary (OT_10_; *n* = 14 sites). Right: Median difference between SI values (OT_10_) = −0.18 (white line). ‘*’: *p* = 1 ×10^−4^, two-sided sign rank test. **F** Recovery (summary). SIs at a subset of OT_10_ sites (*n* = 13), in baseline, OT_10_ GABA blockade, and recovery conditions. ‘*’: significant; two-sided sign rank test, see text for values. **G**–**J** Bar length responses at Imc sites that were spatially aligned to the OT_10_ sites in (**C**–**F**); conventions as in (**C**–**F**). **G** Raster plots of example Imc site (distance of its RF center from OT_10_ RF center in (C) = 1.74°). **H** Firing rate responses (peak normalized) of site in G. SI = 0.52 (baseline), 0.15 (aligned OT_10_ GABA blockade), ‘*’: *p* = 0, one-sided permutation test). Other conventions as in Fig. 2C. **I** Population summary (Imc); average distance between Imc and OT_10_ RF centers = 4.21° ± 1.32°. Conventions as in (**E**). Right: Median difference between SI values (Imc) = −0.2 (white line). ‘*’: *p* = 1 × 10^−4^, two-sided sign rank test. **J** Recovery (summary). SIs at a subset of Imc sites in baseline, OT_10_ GABA blockade, and recovery conditions (*n* = 13 Imc sites spatially aligned with OT_10_ sites from (**F**)). ‘*’: statistically significant; two-sided sign rank test, see text for *p*-values. See also Fig. S3. Source data are provided as a Source Data file.

Since in-situ GABA blockade did not alter the classical inhibitory surrounds of Imc neurons, we next investigated an alternate mechanism for their construction. Specifically, we considered if Imc classical inhibitory surrounds simply reflect the classical inhibitory surrounds of the neurons providing excitatory input to them. Neurons in layer 10 of the OT (OT_10_) provide focal input to Imc neurons, and are the only known source of excitatory drive to them^14,21^. We, therefore, tested if Imc classical surrounds reflect those of OT_10_, by performing paired recordings of classical inhibitory surrounds at spatially aligned Imc and OT_10_ sites, without and with iontophoresis of bicuculline onto OT_10_ neurons (Fig. 3A; Methods).

To this end, we recorded bar length response profiles at paired Imc-OT_10_ sites (Fig. 3B). We first examined the effect of OT_10_ GABA blockade on bar-length response profiles in OT_10_ (Fig. 3C–F, Methods). At an example OT_10_ site, following the application of the GABA blockade, we found a significant decrease in the SI (Fig. 3C, D, SI = 0.36 (baseline), 0.04 (OT_10_ GABA blockade), *p* = 0.002; permutation test), indicating weakening of the classical inhibitory surround in OT_10_. Across a population of OT_10_ sites, we found that GABA blockade consistently reduced SI (Fig. 3E, median = −0.18, *p* = 1 × 10^−4^, sign rank test, *n* = 14 sites). Notably, SI values returned to near-baseline following recovery from bicuculline iontophoresis (Fig. 3F, *p* = 2 ×10^−4^, baseline vs OT_10_ GABA blockade; *p* = 2 × 10^−4^, OT_10_ GABA blockade vs recovery; *p* = 0.03, baseline vs recovery, paired sign rank tests corrected for multiple comparisons, *n* = 13 sites at which recovery was also tested). Thus, GABA blockade in OT_10_ weakened the classical inhibitory surrounds of OT_10_ neurons.

Next, we examined the effect of this OT_10_ GABA blockade on bar-length response profiles at paired (spatially aligned) Imc sites (Fig. 3G–J; Methods). At an example Imc site (the Imc site recorded simultaneously with the example OT_10_ site in Fig. 2C, D, distance between Imc and OT_10_ RF centers = 1.74°), we also found a significant decrease in the SI value following GABA blockade at the paired OT_10_ site, indicating weakening of Imc’s classical inhibitory surround (Fig. 3G, H, SI = 0.52 (baseline), 0.15 (aligned OT_10_ GABA blockade), *p* = 0; permutation test). Across a population of paired (aligned) Imc-OT_10_ sites, we found that GABA blockade at OT_10_ consistently reduced SI at Imc sites (Fig. 3I, average distance between Imc and OT_10_ RF centers = 4.21° ± 1.32°; median change in SI = −0.2, *p* = 1 × 10^−4^, sign rank test, *n* = 14 sites). Indeed, SI values returned to near-baseline values following recovery from OT_10_ bicuculline iontophoresis, demonstrating that the observed effects on SI were due to the drug (Fig. 3J, *p* = 2 × 10^−4^, baseline vs OT_10_ GABA blockade; *p* = 2 × 10^−4^, OT_10_ GABA blockade vs recovery; *p* = 0.13, baseline vs recovery, sign rank tests corrected for multiple comparisons, *n* = 13 sites). Thus, GABA blockade in OT_10_ also weakened the classical inhibitory surrounds of Imc neurons.

Separately, we found that spontaneous firing rates of OT_10_ sites increased upon GABA blockade at OT_10_ and decreased after recovery from the drug (Fig. S3A), consistent with GABA blockade disrupting classical inhibitory surrounds at OT_10_. Notably, we found that spontaneous firing rates at aligned Imc sites also changed in this way following blockade at OT_10_: increase after blockade and decrease after recovery (Fig. S3B).

Taken together, these findings established that inhibition onto, and computations at, OT_10_ are responsible for the expression of classical inhibitory surrounds of Imc neurons, rather than inhibition onto Imc neurons themselves.

### Imc’s extraclassical inhibitory surrounds are constructed using inhibition from other (distant) Imc neurons

**Fig. 4:**
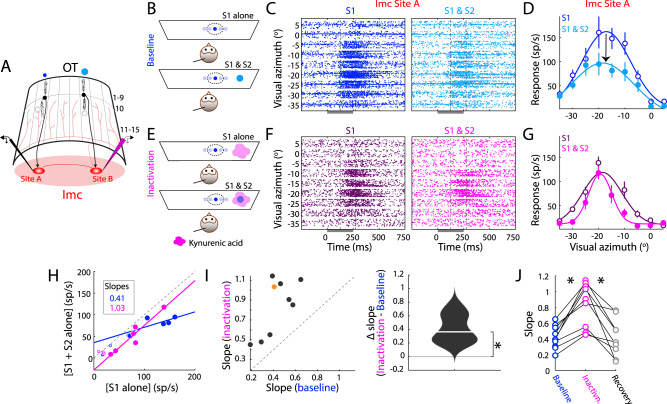
Imc’s extraclassical inhibitory surrounds are constructed using inhibition from other (distant) Imc neurons. **A** Imc-OT schematic showing a tungsten electrode (black) at Imc site A and a glass iontophoretic electrode (pink, kynurenic acid, for silencing excitatory synaptic drive) at a distant Imc site B. Other conventions as in Fig. 1B. **B–D** Baseline condition: Measuring extraclassical inhibitory surrounds of Imc neurons. **B** Stimulus protocol: Spatial tuning curve at Imc site A without (Top) and with a distant competitor (Bottom; S2 > 30° away from site A’s RF center and encoded by Imc site B). **C** Raster responses of example Imc site A. RF center: (−16.41° azimuth, 36° elevation); S1 loom speed = 6°/s. S2 location: (−45° azimuth, 48° elevation); S2 loom speed = 10°/s. Negative azimuths => locations in left hemifield, contralateral to this recording site. Other conventions as in Fig. 1D. **D** Response firing rates (mean ± s.e.m) from (**C**). Other conventions as in Fig. 1E. **E**–**G** Imc site B inactivation condition: Same as (**B**–**D**), but during kynurenic acid iontophoresis (E, pink blob) at Imc site B. (F,G) Responses of Imc site A (from panel **C**) during baseline (purple, filled) and site B inactivation (pink, open) conditions. Distance between site A and site B RF centers = 31°. **G** Response firing rates (mean ± s.e.m) from (**F**). **H** Scatter plots of responses of the example Imc site A. Blue: baseline (from **D**). Pink: site B inactivation (from **G**). Slopes of best fit lines: 0.41 (baseline), 1.03 (site B inactivation), *p* = 0.034, one-sided permutation test. Other conventions as in Fig. 1I. **I** Population summary. Left: Site B inactivation vs. baseline slopes (*n* = 9 site pairs); distance between Imc site A and site B RF centers = 44.7° ± 5.17°. Orange dot: example site shown in (**C**–**G**). Right: Violin plot of difference in slopes; median =0.36 (white line). ‘*’: *p* = 0.004, two-sided sign rank test. **J** Recovery (summary). Slopes (*n* = 9 site pairs), measured in baseline, kynurenic acid, and recovery conditions. ‘*’: significant; see text for test and *p*-values. See also Fig. S4. Source data are provided as a Source Data file.

Contrary to classical inhibitory surrounds of Imc neurons, Fig. 1 showed that their extraclassical surrounds are interrupted by blockade of inhibition onto the Imc neurons. We were next interested in identifying the source of this inhibition. Past work in slice has revealed the presence of long-range inhibitory projections between Imc neurons^14,26^. To test if inhibition from Imc neurons encoding for distant locations controls the construction of extraclassical (competitive) surrounds in Imc, we conducted paired recordings at two mutually distant sites within the Imc (Fig. 4A and S4; Methods). Specifically, using the same stimulus protocol as in Fig. 1 (Fig. 1A, B), we recorded tuning curve responses at one Imc site (site A) using stimulus S1, while simultaneously presenting S2 at a location > 30° away, encoded by distant Imc neurons with non-overlapping spatial receptive fields (site B; Fig. 4B)^19,20,27^. We then compared responses at Imc site A without and with iontophoretic silencing of Imc site B using kynurenic acid (Fig. 4B, E; Methods).

We found that in the baseline condition (Fig. 4B), S2 effectively suppressed S1 tuning curve responses recorded at site A (Fig 4C, D, H; example site, slope = 0.41). Notably, focally inactivating Imc site B (distant site encoding the competitor stimulus S2; Fig. 4E, Fig. S4A–C), abolished S2’s suppressive effect on responses of Imc site A (Fig. 4F–H; slope=1.03; *p* = 0.034, permutation test of site B inactivation vs. baseline slopes). This effect was consistent across the population of tested site pairs (Fig. 4I-left, *n* = 9 site A-site B pairs): inactivation of Imc site B significantly weakened S2-induced suppression of S1 responses at Imc site A (Fig. 4I-right; median slope increase of 0.36; *p* = 0.004, sign rank test of difference in slopes at site A in baseline vs. site B inactivation conditions).

The observed effects were due, specifically, to drug iontophoresis. First, evoked responses at site B, which were inactivated by kynurenic acid (Fig. S4A, B), returned to near baseline levels following recovery from kynurenic acid (Fig. S4C). Second, S2-induced suppression of S1 responses at site A, which was nearly abolished during kynurenic acid inactivation of site B, also returned to near-baseline levels following recovery (Fig. 4J; *p* = 0.004, baseline vs. site B inactivation; *p* = 0.004, site B inactivation vs. recovery; *p* = 0.82 baseline vs. recovery, paired sign rank tests corrected for multiple comparisons).

We were able to rule out the possibility that the observed effects were due to potential spread of kynurenic acid from Imc site B to Imc site A. We have shown previously that iontophoresis of kynurenic acid at an Imc site does not block evoked responses at distant Imc sites whose RFs are located more than 25° away^16^, and for this reason, in our experiments here, A and B sites were chosen to be much further apart (average distance = 44.7° ± 5.17°). To test any effects of potential spread directly, we performed separate experiments in which A and B sites in Imc were similarly far apart (average distance between Imc site A and site B RF centers = 45.7° ± 6.54°; *n* = 5; Fig. S4D–F). We confirmed that kynurenic acid iontophoresis at B sites did not spread to A sites: maximum evoked responses at A sites were largely unchanged between baseline and kynurenic acid conditions (Fig. S4D: *p* = 0.37, t-test between peak firing rates; Fig. S4F, site A: *p* = 0.63, Kruskal–Wallis test followed by correction for multiple comparisons), unlike at B sites at which they were suppressed significantly (Fig. S4E: *p* = 4 × 10^−9^, t-test between peak firing rates; Fig. S4F, site B: *p* = 0.036, Kruskal–Wallis test followed by correction for multiple comparisons).

Together, these results established that extraclassical inhibitory surrounds of Imc neurons are constructed at the Imc using long-range inhibition from distant Imc neurons, i.e., intra-Imc inhibition is the source of Imc’s extraclassical inhibitory surrounds.

## Discussion

This study uncovers the mechanistic implementation of classical as well as global inhibitory surrounds of neurons in the Imc, a key midbrain inhibitory nucleus in the tecto-fugal pathway for visuomotor (and more generally, sensorimotor) processing in vertebrates^8^. In the parallel thalamo-cortical pathway of visual processing, a related debate on whether the classical surrounds of simple cells in V1^28^ are constructed within V1 by the action of local inhibitory neurons, or by modulation of excitatory inputs from upstream LGN cells, was only recently resolved^29^. Selective manipulation of inhibitory neurons within V1 in mice showed that cortical inhibition played a key role in the construction of the classical surround^30^, thereby establishing a clear mechanism for a basic function of V1 neurons.

In the tecto-fugal pathway, the sensorimotor hub, SC/OT, is not only involved in the processing of individual sensory stimuli, but also plays a critical role in the selection of the highest priority stimulus (target for spatial attention) among competing distracters^5,6,31^. In turn, neurons in the inhibitory Imc control the competitive interactions within OT^15,16^. This inhibitory output of Imc neurons reflects computations occurring within Imc rather than being just a sign-flipped version of excitatory inputs into Imc^19,20^. Indeed, the Imc itself encodes a map of relative stimulus priority^19,20^, much like the SC/OT – the first reported in inhibitory neurons, to the best of our knowledge. In this context, then, how the responses of Imc neurons to (single and multiple competing) stimuli are constructed, and specifically, how Imc receptive fields are constructed, is a critical, but unanswered, question.

Past work involving recordings in the Imc paired with focal iontophoretic silencing of OT_10_ neurons have clearly identified OT_10_ as the source of Imc’s excitatory drive^21^. However, although that study also examined the effect of iontophoretic blockade of GABAergic input in Imc on Imc responses^21^, the results are difficult to interpret in the context of the construction of Imc’s inhibitory surrounds. In that study, data obtained from the presentation of a single stimulus inside the RF, and from the presentation of two competing stimuli (one inside and one outside the RF), were combined and reported together as a single result. Classical versus global (extraclassical) surrounds have fundamentally different properties in terms of their function, strength profiles, spatial scope, and requirements of the underlying circuitry^27^. As a result, the properties of one cannot predict those of the other^27^, highlighting the critical need for considering results from single stimulus versus two-stimulus (competition) protocols separately in order to disambiguate the mechanistic underpinnings of the respective inhibitory surrounds.

Doing so, here, revealed that stimulus competition is computed within the Imc via inhibitory projections among Imc neurons: these long-range projections^14,26^ create global inhibitory surrounds of Imc RFs (Figs. 1, 4). By contrast, classical inhibitory surrounds of Imc RFs are not computed within the Imc (Figs. 2, 3). These latter findings are consistent with the observation in midbrain slice experiments that Imc neurons do not appear to receive projections from nearby (spatially “local”) Imc neurons^26^. Our results showed, instead, that the classical inhibitory surrounds of Imc RFs are inherited entirely via the excitatory input from OT_10_ neurons. Indeed, consistent with this finding, we show that spontaneous rates in Imc do increase significantly upon iontophoresis of bicuculine in OT_10_ (Fig. S3C, D).

An intriguing question that arises from these findings is, ‘why might the Imc-OT circuit be organized this way?’ In other words, why doesn’t the Imc inherit both classical and global inhibitory surrounds from the OT, or alternatively, why doesn’t it compute both locally? A plausible answer is offered by the fundamental function of the Imc, namely, the orchestration of stimulus selection in the midbrain attention network, and specifically, in the OT.

Imc is the dominant source of competitive inhibition to the intermediate and deep layers of the OT (OTid)^15,16^, is necessary for the OTid to signal the highest priority stimulus^15^, controls selection at all possible pairs of locations in the OTid space map through an optimized combinatorial inhibition solution^17^, drives categorical stimulus selection across the OTid space map^32^, and is thought to be critical for mediating flexibility of selection boundaries in the OT^33^. Additionally, Imc itself expresses signatures of global stimulus competition across space, and does so earlier than the OTid^19,20^. Consequently, having a dedicated circuit mechanism within the Imc that can implement extraclassical inhibitory surrounds and achieve stimulus competition, would be beneficial.

By contrast, Imc neurons have been shown to not send inhibition to the portion of the OT space map from which they receive input (referred to a donut-like spatial inhibition^14,32^. Due to this specialized anatomical and functional feature, Imc does not participate in shaping OTid’s responses to single stimuli. Consequently, not having a dedicated mechanism within the Imc for generating classical inhibitory surrounds (which typically help shape responses to single stimuli) avoids potentially ‘wasteful’ circuitry that would not aid the core functional role of Imc.

In other words, the computation of global surrounds, but inheritance of classical surrounds, in the Imc may represent an efficient circuit implementation for stimulus selection in the midbrain spatial attention network.

## Methods

### Experimental design

The goal of this study was to determine the mechanisms underlying the construction of classical and extraclassical inhibitory surrounds of Imc neurons. This was done by measuring (i) spatial tuning curves at Imc neurons using a visual stimulus (S1, presented at various azimuthal locations within and immediately outside the receptive field, RF), (ii) with or without a second visual stimulus present outside of the RF (S2), and separately, measuring (iii) responses to bar stimuli of increasing lengths. All these measurements were made in baseline conditions, and while microiontophoretically applying bicuculline methiodide (Sigma-Aldrich), a GABA_A_ receptor antagonist locally at the recording site. This allowed us to compare the effect of GABAergic input on local surrounds (S1 tuning curves and bar length response profiles), and as well, on competitive surrounds (S1 tuning curves when S2 was also presented simultaneously).

Additionally, we examined the role of OT_10_ neurons in the construction of classical inhibitory surrounds of Imc neurons by applying bicuculline methiodide at OT_10_ sites while simultaneously recording tuning curves and bar length response profiles at both the OT_10_ site and a paired Imc site encoding for the same area of sensory space (spatially aligned site). Further, to examine the role of Imc neurons in mediating the extraclassical surrounds of other (distant) Imc neurons, we recorded from one Imc site encoding for S1 (site A) without and with S2 presented far outside of the site’s RF (>30° away). A second electrode, placed at the distant Imc site encoding for S2 (spatially misaligned site B), was then used to inactivate Imc site B (using kynurenic acid). We compared the responses of Imc sites A in the intact versus site B-inactivated cases. Additionally, in control experiments to assess potential spread of kynurenic acid iontophoresis at site B to site A, we compared the responses of Imc sites A in the intact versus site B-inactivated cases while recording spatial receptive fields at both sites.

### Neurophysiology

Eight adult barn owls (Tyto alba; no specific sex selection) were used for electrophysiological recordings. All protocols and animal care were in accordance with NIH guidelines for care and use of laboratory animals and approved by the Johns Hopkins University Institutional Animal Care and Use Committee. Birds were shared across different studies and group housed in an aviary with a 12 h/12 h light/dark cycle. Before electrophysiological experiments, head bolts were affixed to the skull under anesthesia (isoflurane, 1–2%, and a mixture of nitrous oxide and oxygen, 45:55). Birds were administered intramuscular injections of 0.1 mL of meloxicam and 0.1 mL of butorphanol, incision areas were disinfected with betadine, and locally anesthetized with subcutaneously injected bupivacaine. Bilateral craniotomies were performed and small plastic cylinders with removable caps were placed on the skull to allow access to midbrain structures over multiple experiments. Polysporin antibiotic ointment was applied to any exposed brain surface and incisions. Owls were returned to the aviary following recovery from surgery, and experiments were performed starting after a week of recovery.

On experiment days, owls were initially anesthetized with isoflurane (1–2%) and a mixture of nitrous oxide and oxygen (45:55), and administered with intramuscular injections of 0.1 mL of meloxicam and 0.1 mL of butorphanol. Birds were then secured in a sound-attenuating booth, and head-fixation was calibrated for each owl as follows. The pupils were dilated with atropine eye drops, and the pectin structures in the eye were sighted using an ophthalmoscope. The position of the head of the owl was adjusted in roll, pitch and yaw directions such that the pectin structures in the two eyes were positioned symmetrically on either side of the midline (~25 visual degrees from midline), and positioned approximately 7 degrees above horizon^34^.

Once the bird was calibrated, isoflurane was turned off after the bird was secured, and owls were maintained on oxygen and nitrous oxide for the duration of the experiment. As recovery from isoflurane occurs well under 30 min after it is turned off, recordings were made in animals that were not anesthetized. When possible, nitrous oxide as well was turned off 5 min before data collection (it partitions out of blood rapidly, within a minute). Notably, previous work has demonstrated that neural responses in the midbrain network do not differ under nitrous oxide tranquilization from non-tranquilized conditions^11^.

The Imc is an oblong structure in the avian midbrain that is elongated along the rostrocaudal axis, parallel to the OT. Previous work has confirmed in vivo targeting of the Imc with fluorescent dye injection^16^ and with electrolytic lesions^17^, and established its location as approximately 500 μm medial to the medial-most part of the OT. Recording sites in the Imc were targeted by either navigating first to the optic tectum and then to the Imc using the OT’s topographic space map as ref. ^20^, or by referencing reliable stereotaxic coordinates from prior experiments and verified on the basis of established distinct neural responses^11,16,17,20,27^. For recording in OT_10_, OT layers were identified by their distinctive neuronal responses^34^.

### Recordings

For Imc recordings, an electrode was positioned to enter the brain at a medial-leading angle of 5° to avoid a major blood vessel. During some paired Imc-Imc recordings, electrodes were additionally angled in the caudal-leading direction (2°−5°) to accommodate space for two electrodes. For paired OT_10_-Imc recordings, the OT_10_ targeting electrode was also angled at a 5° medial-leading angle to sterically accommodate both OT_10_ and Imc electrodes. Extracellular activity in Imc was recorded primarily using multi-barreled glass electrodes with the central barrel containing a carbon fiber electrode for recording neural activity (Kation, Carbostar-3 Carbon fiber electrode). Paired recordings with two electrodes utilized a multi-barreled glass electrode to administer drug and record responses at one brain site, and an epoxy-coated tungsten microelectrode (A-M Systems, 5 MΩ at 1 kHz, 250 μm shaft diameter) to record responses at the paired site. All data in this paper represent a combination of both well-isolated single as well as multi-unit sites. Spike times were recorded using Tucker-Davis hardware and analyzed using MATLAB.

### Microiontophoresis

Microiontophoresis was performed using a 1-channel iontophoresis box (DAGAN Corp PS-100). This is an established technique for focal delivery of drugs, and has been used extensively for this purpose in the literature^32,34^. The drug of interest was filled in one barrel of a multi-barreled electrode and was microiontophoretically applied to the recording site. Electrophysiological responses were recorded using a carbon fiber electrode in one of the other barrels.

To achieve blockade of inhibitory synaptic input, the GABA_A_ antagonist bicuculline methiodide (Sigma, 10 mM, 2.37–2.67 pH, mean pH = 2.54) was used. For bicuculline iontophoresis in OT_10_, we used ejection currents based on published work that has used bicuculline iontophoresis in owl OT (80 nA;^35,36^. For bicuculline iontophoresis in Imc, we used ejection currents in the range of 20–80 nA (42.5 nA ± 2.52 nA; *n* = 44 sites). This was guided by the ranges in published work: 50–100 nA in Imc^21^, 30–120 nA in OT^35,36^, 20–80 nA in other regions of the central nervous system of several species^37–41^, and pilot experiments. Bicuculline was ejected at Imc or OT sites for 15 min before data collection in the drug condition and ceased for 25–35 min before data collection in the recovery condition. When not being ejected, bicuculline was retained at a current of −15nA.

To achieve focal blockade of excitatory synaptic input in Imc, the pan-glutamate receptor antagonist kynurenic acid (Sigma, 40 mM, 8.5-9 pH) was used. For kynurenic acid iontophoresis (in Imc), we used ejection currents established in published work (−500nA)^16^. Kynurenic acid was ejected for 15 min before data collection in the drug condition and ceased for 15 min before data collection in the recovery condition^16^. When not being ejected, Kynurenic acid was retained at +15 nA.

Microiontophoretic experiments typically involved three conditions: baseline, drug delivery (GABA or Kynurenic acid) and recovery. By necessity, they are performed serially. The time between the start of “baseline” data collection and the start of “drug” data collection is ~ 45 min (data collection in each condition takes approximately 30 min, with ~15 min wait time in between, for the drug to take effect). Similarly, the time between the start of “drug” data collection and the start of “recovery” data collection is ~ 45–60 (30 min data collection + ~15–30 min wait time for drug’s effect to wear off). Such extended electrophysiological recording sessions are known to often result in a progressive run-down of responses over time, which we also observed in our experiments (Figs. S1, S2).

### Stimuli

Stimuli were black dots (looming stimuli) or black horizontal bars (stationary stimuli) on a gray background presented on a 65” monitor placed tangentially at a distance of 12.5” in front of the owl. The looming dot stimuli expanded linearly over a duration of 250 ms to mimic approaching objects; previous work has estasblished that looming dot stimuli evoke reliably strong responses in OT and Imc with relatively low response habituation^11,27^. Bar stimuli were of fixed height (3°), but varying lengths. Stimuli were presented using MATLAB and Psychtoolbox (PTB-3^42,43^). The spatial locations of visual stimuli were defined by double pole coordinates relative to the midsagittal plane for azimuth or the visual plane for elevation^34^.

To determine the extent of space encoded by a recorded site (in Imc or OT_10_), we collected two-dimensional RFs (azimuth × elevation) by presenting a looming dot stimulus at various azimuthal and elevational locations. Spatial locations at which the stimulus elicited higher firing rates compared to baseline were defined as the site’s spatial RF. This was used for determining the placement of S1 (within the RF), of S2 (outside the RF), and for determining the alignment of Imc and OT_10_ sites.

Tuning curves were measured by presenting S1 of a fixed strength (6°/s) at multiple azimuthal locations spanning the extent of a site’s RF. For examining extraclassical surrounds, S2 was presented far outside the RF, at a distant location from S1 (mean distance from RF center = 33.3 ± 0.91°). S2 loom speed (10°/s), S2 was stronger than of S1. Presentations of S1 alone, and S1 with S2, were interleaved pseudo randomly. Stimuli were presented for 15 repetitions for collecting the data in Figs. 1–4. For examining classical inhibitory surrounds, bar-length response functions were obtained by presenting horizontal bars of different lengths (typically, 0.5° to ~40°) centered within the RF. Trials of the three types (tuning curves with S1 alone, tuning curves with S1 in the presence of a distant S2, and bar length response functions) were interleaved randomly. In all cases, stimuli were presented for 15 repetitions, with a duration of 250 ms each and an interstimulus interval of 1500 ms.

### Data analysis

All analyses were done with MATLAB scripts. Response firing rates were determined by counting the number of spikes over a time window following stimulus onset and converting this count to firing rate (sp/s) after subtracting the baseline firing rate. This window was chosen to capture well the evoked neural responses: for tuning curves, the count window was from 125 ms to 275 ms with respect to stimulus onset (for Imc^19^, and 100 ms to 250 ms (for OT_10_^11,26,27,33^). For bar length response curves, the count window was from 65 ms to 175 ms (for both OT_10_ and Imc sites^27^). Average rates were calculated across all presentation repetitions.

### Statistics

All statistical analyses were performed in MATLAB. Outliers were identified as data points that lay outside the range of median ± 1.5*interquartile range of the distribution. Parametric tests (ANOVA, t-tests) were used if data were normally distributed (tested using a Kolmogorov-Smirnov test, *kstest*), non-parametric tests (Kruskal-Wallis, sign rank) were used otherwise. Correction for multiple comparisons was performed using the Holm-Bonferroni method (for sign rank tests,) and Dunn-Sidak for Kruskal-Wallis, if needed. All tests were two-sided. Data shown as a ± b refer to mean ± s.e.m, unless specified otherwise. For bar length response functions, the suppression index (SI), was defined as the difference between the peak response and the asymptotic response (estimated as the average response to three widest bars) divided by the peak response.

The permutation test comparing the SI values of bar length response profiles between baseline and GABA blockade conditions (at the example sites in Figs. 2, 3) was performed as follows: First, we randomly assigned firing rates measured at each bar length (15 repetitions × 2 conditions) to either the baseline or GABA blockade condition. Following this, we computed the mean responses (at each bar length), and then the ‘shuffled’ suppression index (‘shuffled’ SI), for each condition, and calculated the difference in shuffled SIs. This procedure was repeated 500 times to generate a distribution of ‘shuffled’ SI differences, and the actual difference in SI was compared to this shuffled null distribution. The p-value was calculated as the proportion of permutations that resulted in a difference greater than or equal to the site’s actual difference of ratios.

The permutation test comparing the slopes of competitive suppression between baseline and GABA blockade conditions (at the example sites in Figs. 1, 4) was performed as follows: First, we randomly assigned firing rate-pairs (for the S1 and S1&S2 presentations) measured at each S1 location (15 repetitions × 2 conditions) to either the baseline or GABA blockade condition. Following this, we computed the mean responses (at each S1 location), and the ‘shuffled’ slope, for each condition, and calculated the difference in shuffled slopes. This procedure was repeated 500 times to generate a distribution of ‘shuffled’ slopes, and the actual difference in slopes was compared to this shuffled null distribution. The p-value was calculated as the proportion of permutations that resulted in a difference greater than or equal to the site’s actual difference of ratios.

For paired Imc-Imc recordings, site-pairs were included only when kynurenic acid iontophoresis caused significant reduction of responses at site B.

### Reporting summary

Further information on research design is available in the Nature Portfolio Reporting Summary linked to this article.

## Supplementary information

Supplementary Information

Reporting Summary

## Data Availability

Data supporting the findings of this study are available in the Zenodo repository^44^: 10.5281/zenodo.7827183 Source data are provided as a Source Data file. Source data are provided with this paper.

## Source data

Source Data

## Code availability

The software code that supports the findings of this study are available in the Zenodo repository^44^: 10.5281/zenodo.7827183.
