# Supplementary material for: Distinct neural mechanisms construct classical versus extraclassical inhibitory surrounds in an inhibitory nucleus in the midbrain attention network
Source: Nat Commun. 2023 Jun 9;14:3400. doi: 10.1038/s41467-023-39073-5 (PMC10256684; doi:10.1038/s41467-023-39073-5)
Supplement: Supplementary file 1 — Supplementary Information [file 41467_2023_39073_MOESM1_ESM.docx]

Supplementary Information

Distinct neural mechanisms construct classical versus extraclassical inhibitory surrounds in an inhibitory nucleus in the midbrain attention network

**Hannah M. Schryver^1,2^ and Shreesh P. Mysore^1,3,4*^**

*^1^Department of Psychological and Brain Sciences, Johns Hopkins University, Baltimore MD 212182*

*^2^Currently, Allen Institute, Seattle, WA*

*^3^The Solomon H. Snyder Department of Neuroscience, Johns Hopkins School of Medicine, Baltimore MD 21205*

*^4^Kavli Neuroscience Discovery Institute, Johns Hopkins University, Baltimore MD 21218*

**Submitting and corresponding author:* [*mysore@jhu.edu*](mailto:shreesh.mysore@jhu.edu)

**
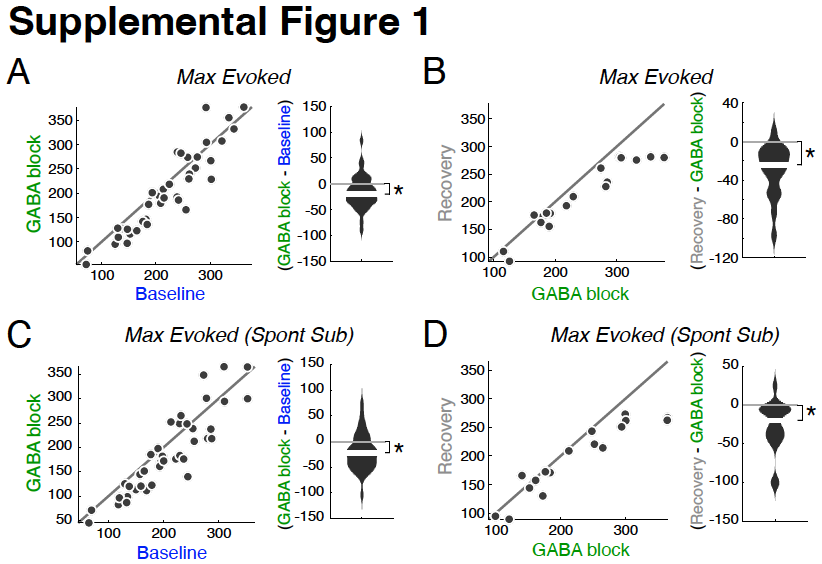
**

**Supplemental Figure 1. Maximum firing rates for Imc sites in Figure 1.**

**(A)** Left: Scatter plot of max evoked firing rates in baseline versus GABA blockade conditions (n=40). Right: Violin plot of difference in firing rates in GABA blockade and baseline conditions for each site. Gray line indicates zero difference; white line indicates median difference (-19.78 sp/s). ‘*’: statistically significant; p=0.0015, sign rank test.

**(B)** Left: Scatter plot of max evoked firing rates in GABA blockade vs recovery conditions at those sites where recovery data was collected (n=18). Right: Violin plot of difference in firing rates in recovery and GABA blockade conditions for each site. Gray line indicates zero difference; white line indicates median difference (-24.22 sp/s). ‘*’: statistically significant; p=4 x 10^-4^, sign rank test.

**(C,D)** Same as (A,B), except that max evoked firing rate at each site was calculated after subtraction of spontaneous rate at that site (the standard procedure in this study). (C) Baseline versus GABA blockade; median difference = -21.81 sp/s. ‘*’: statistically significant; p=0.0012, sign rank test. (D) GABA blockade vs recovery; median difference = -19.77 sp/s. ‘*’: statistically significant; p=9 x 10^-4^, sign rank test. All other conventions as in A,B. Source data are provided as a Source Data file.

**
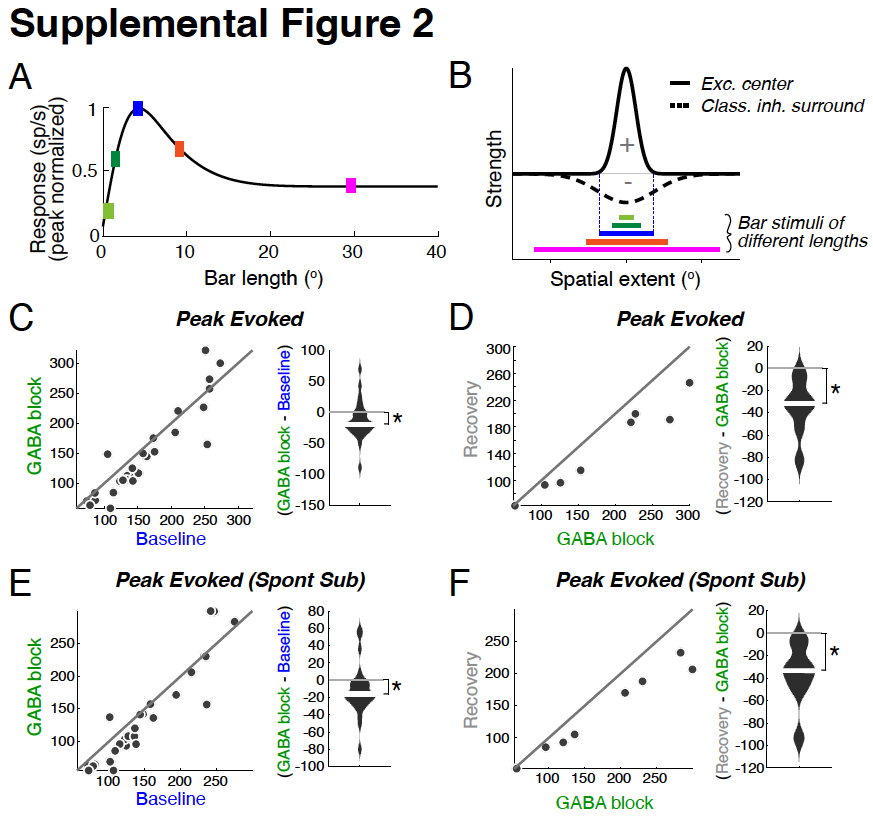
**

**Supplemental Figure 2. Peak evoked firing rates for Imc sites in Figure 2.**

**(A)** Schematic of a classic bar-length response profile. Colored boxes indicate in sequence: weak responses at small bar length (light green), increasing responses for medium bar length (dark green), peak responses (blue), falling response for longer bar length (orange), and asymptotic response for very long bar lengths (pink).

**(B)** Schematic of center-surround structure of spatial receptive field, with strong but narrow excitatory center (+; solid line) and weaker but broader classical inhibitory surround (-; dashed line), that produces classic bar length response profile in E. Shown also are schematic bar lengths that produce the responses of the five different kinds (colored boxes) in E.

**(C)** Left: Scatter plot of peak evoked firing rates in baseline versus GABA blockade conditions (n=28); Methods). Right: Violin plot of difference in firing rates in GABA blockade and baseline conditions for each site. Gray line indicates zero; white line indicates median difference (-19.25 sp/s). ‘*’: statistically significant; p=0.0095, sign rank test.

**(D)** Left: Scatter plot of peak evoked firing rates in GABA blockade vs recovery conditions; (only sites where recovery was collected, n=8). Right: Violin plot of difference in firing rates in recovery and GABA blockade conditions for each site. Gray line indicates zero; white line indicates median difference (-31.64 sp/s). ‘*’: statistically significant; p=0.0078, sign rank test.

**(E,F)** Same as (C,D), except that max evoked firing rate at each site was calculated after subtraction of spontaneous rate at that site (the standard procedure in this study). (E) Baseline versus GABA blockade; median difference = -16.03 sp/s. ‘*’: statistically significant; p=0.0059, sign rank test. (F) GABA blockade vs recovery; median difference = -33.37 sp/s. ‘*’: statistically significant; p=0.0078, sign rank test. All other conventions as in A,B. Source data are provided as a Source Data file.

**
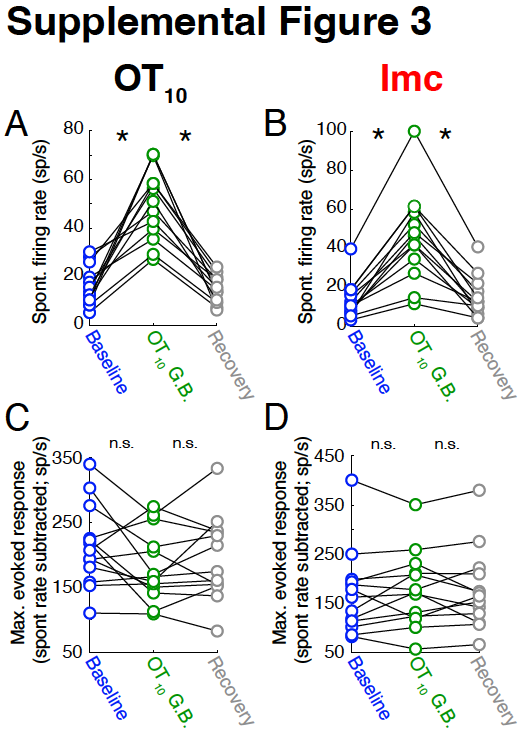
**

**Supplemental Figure 3. Spontaneous firing rate for OT_10_ and Imc sites in Figure 3.**

**(A)** Plot of spontaneous firing rates (in spikes per second) measured at OT_10_ sites in Figure 3 (n=13), in the baseline, OT_10_ GABA blockade, and recovery conditions. ‘*’: statistically significant (Baseline vs inactivation: p=2 x 10^-4^. Inactivation vs recovery: p=2 x 10^-4^. Baseline vs recovery: p= 0.8. All sign rank tests followed by Holm-Bonferroni correction for multiple comparisons).

**(B)** Plot of spontaneous firing rates (in spikes per second) measured at paired Imc sites in Figure 3 (n=13), in the baseline, OT_10_ GABA blockade, and recovery conditions. ‘*’: statistically significant (Baseline vs inactivation: p=2 x 10^-4^. Inactivation vs recovery: p=2 x 10^-4^. Baseline vs recovery: p=0.35. All sign rank tests followed by Holm-Bonferroni correction for multiple comparisons).

**(C)** Plot of maximum evoked firing rates (after subtraction of spontaneous firing rates) measured at OT_10_ sites in Figure 3 (n=13 sites), in the baseline, OT_10_ GABA blockade, and recovery conditions. ‘ns’: not significant; sign rank test, p<0.05 (Baseline vs inactivation: p=0.147. Inactivation vs recovery: p=0.455. Baseline vs recovery: p= 0.191.)

**(D)** Plot of maximum evoked firing rates (after subtraction of spontaneous firing rates) measured at paired Imc sites in Figure 3 (n=13 sites), in the baseline, OT_10_ GABA blockade, and recovery conditions. ‘ns’: not significant; sign rank test, p<0.05 (Baseline vs inactivation: p=0.455. Inactivation vs recovery: p=0.839. Baseline vs recovery: p=0.305.) Source data are provided as a Source Data file.


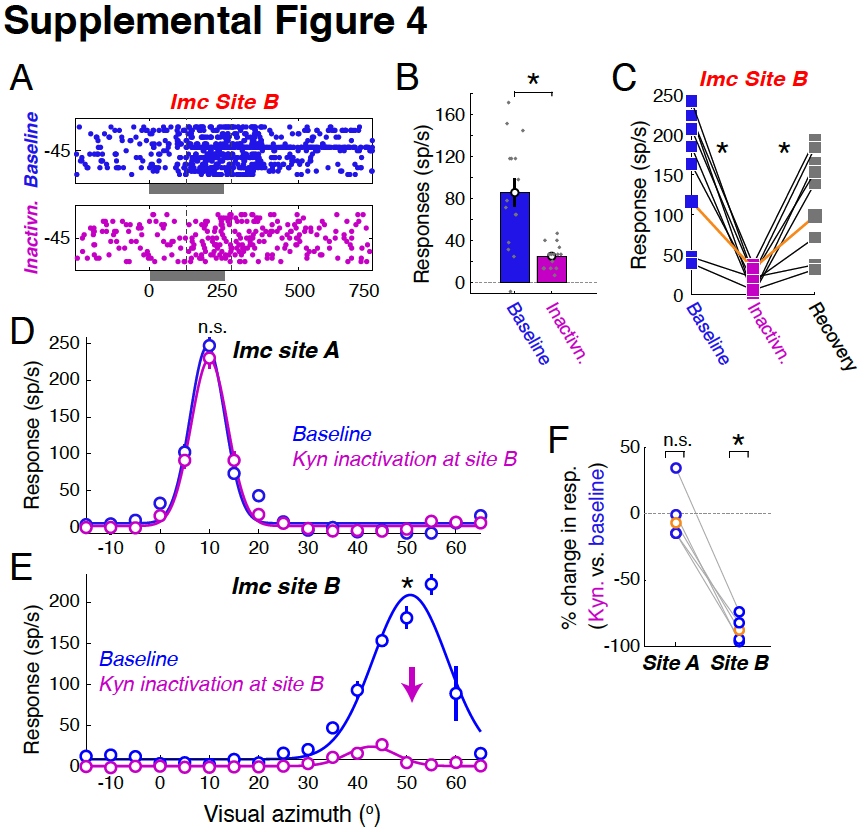


**Supplemental Figure 4. Firing rates for Imc sites B (Fig. 4), and control experiments.**

**(A)** Raster responses of example Imc site B to S2 (-45° azimuth, 48° elevation) while S1 was also presented around -15° azimuth, 36° elevation; responses recorded simultaneously with example site in Fig 4C-G. Top: Baseline. Bottom: Kynurenic acid iontophoresis at site B (“Imc site B inactivation”; Methods). Other conventions as in Fig 4C,F, respectively.

**(B)** Response firing rates (mean ± s.e.m), from rasters in A. ‘*’: statistically significant; p=4 x 10^-4^; two-sided sign rank test (n=15 repetitions of stimulus presentation).

**(C)** Recovery at Imc site B (summary). Firing rates at all Imc sites B recorded simultaneously with Imc sites A in Figure 4 (n=9), measured in baseline, site B inactivation, and recovery conditions. ‘*’: significant; baseline vs inactivation: p= 0.0039; inactivation vs recovery: p=0.0039; baseline vs recovery: p= 0.0039; two-sided sign rank tests followed by Holm-Bonferroni correction for multiple comparisons. Orange data: example site from panels A and B.

**(D-F)** Separate control experiments to assess potential spread of kynurenic acid iontophoresed at site B to site A, by measuring effects of Imc site B inactivation on Imc site A responses.

(D Tuning curve responses (mean ± s.e.m) measured at example site A. Solid lines: Gaussian fits. Site A RF center (10° azimuth, -25° elevation), stimulus loom speed = 6 °/s. “n.s.”: not statistically significant, p=0.37, baseline vs. Imc site B inactivation, two-sided t-test between firing rates (at azimuth = 10°).

(E) Tuning curve responses (mean ± s.e.m) measured simultaneously at distant Imc site B; RF center (~50° azimuth, -25° elevation), stimulus loom speed = 6 °/s. “*”: statistically significant, p=4*10^-9^, two-sided t-test between firing rates (at azimuth = 50°).

(F) Summary of effects at Imc site A and site B following kynurenic acid at Imc site B (n=5 site pairs; average distance between Imc site A and site B RF centers = 45.7° ± 6.54°). Grey lines: paired sites; orange dots: from example site in D, E. ‘n.s.’: not statistically significant (site A), ‘*’: significant (site B); Kruskal-Wallis test (χ^2^(2)=10.89, p=0.0043) followed by paired tests with correction for multiple comparisons for site A against 0 (p= 0.63), and site B against 0 (p= 0.036). Source data are provided as a Source Data file.
