# Supplementary material for: Distinct neural mechanisms construct classical versus extraclassical inhibitory surrounds in an inhibitory nucleus in the midbrain attention network
Source: Nat Commun. 2023 Jun 9;14:3400. doi: 10.1038/s41467-023-39073-5 (PMC10256684; doi:10.1038/s41467-023-39073-5)
Supplement: Supplementary file 2 — Reporting Summary [file 41467_2023_39073_MOESM2_ESM.pdf]

Corresponding author(s): Shreesh P. Mysore

Last updated by author(s): 04/13/2023

Reporting Summary

Nature Portfolio wishes to improve the reproducibility of the work that we publish. This form provides structure for consistency and transparency in reporting. For further information on Nature Portfolio policies, see our Editorial Policies and the Editorial Policy Checklist.

Statistics

For all statistical analyses, confirm that the following items are present in the figure legend, table legend, main text, or Methods section.

n/a	Confirmed	
☐	☒	The exact sample size (*n*) for each experimental group/condition, given as a discrete number and unit of measurement
☐	☒	A statement on whether measurements were taken from distinct samples or whether the same sample was measured repeatedly
☐	☒	The statistical test(s) used AND whether they are one- or two-sided<br>*Only common tests should be described solely by name; describe more complex techniques in the Methods section.*
☐	☒	A description of all covariates tested
☐	☒	A description of any assumptions or corrections, such as tests of normality and adjustment for multiple comparisons
☐	☒	A full description of the statistical parameters including central tendency (e.g. means) or other basic estimates (e.g. regression coefficient) AND variation (e.g. standard deviation) or associated estimates of uncertainty (e.g. confidence intervals)
☐	☒	For null hypothesis testing, the test statistic (e.g. F, t, r) with confidence intervals, effect sizes, degrees of freedom and P value noted<br>*Give P values as exact values whenever suitable.*
☒	☐	For Bayesian analysis, information on the choice of priors and Markov chain Monte Carlo settings
☒	☐	For hierarchical and complex designs, identification of the appropriate level for tests and full reporting of outcomes
☒	☐	Estimates of effect sizes (e.g. Cohen's *d*, Pearson's *r*), indicating how they were calculated

Our web collection on statistics for biologists contains articles on many of the points above.

Software and code

Policy information about availability of computer code

Data collection	Multi-unit spike waveforms were recorded using Tucker Davis Technologies hardware interfaced with MATLAB (2022a) and Psychtoolbox-3.
Data analysis	All analyses were carried out using standard MATLAB functions.

For manuscripts utilizing custom algorithms or software that are central to the research but not yet described in published literature, software must be made available to editors and reviewers. We strongly encourage code deposition in a community repository (e.g. GitHub). See the Nature Portfolio guidelines for submitting code & software for further information.

Data

Policy information about availability of data

All manuscripts must include a data availability statement. This statement should provide the following information, where applicable:

- Accession codes, unique identifiers, or web links for publicly available datasets
- A description of any restrictions on data availability
- For clinical datasets or third party data, please ensure that the statement adheres to our policy

Software code and the data that support the findings of this study are available in the Zenodo repository (link provided in the data availability statement). Source data is provided with this paper.

Human research participants

Policy information about studies involving human research participants and Sex and Gender in Research.

Reporting on sex and gender	N/A
Population characteristics	N/A
Recruitment	N/A
Ethics oversight	N/A

Note that full information on the approval of the study protocol must also be provided in the manuscript.

Field-specific reporting

Please select the one below that is the best fit for your research. If you are not sure, read the appropriate sections before making your selection.

☒ Life sciences ☐ Behavioural & social sciences ☐ Ecological, evolutionary & environmental sciences

For a reference copy of the document with all sections, see nature.com/documents/nr-reporting-summary-flat.pdf

Life sciences study design

All studies must disclose on these points even when the disclosure is negative.

Sample size	Sample size calculations were not performed; rather, choices were guided by similar, previously published studies. Iontophoresis experiments (all figures): Mahajan & Mysore (2022; Nature Communications), Mysore & Knudsen (2013; Nature Neuroscience), Asadollahi & Knudsen (2016; Nature Communications). Tuning curve measurements (Figures 1 & 4): Mahajan & Mysore (2022; Nature Communications), Mysore & Knudsen (2013; Nature Neuroscience), Asadollahi & Knudsen (2016; Nature Communications), Mysore et al (2010; Journal of Neuroscience), Schryver & Mysore (2019; Brain, Behavior, & Evolution). Competitive stimulus suppression (Figures 1 & 4): Mahajan & Mysore (2022; Nature Communications), Mysore & Knudsen (2013; Nature Neuroscience), Asadollahi & Knudsen (2016; Nature Communications), Mysore et al (2010; Journal of Neuroscience), Schryver & Mysore (2019; Brain, Behavior, & Evolution). Receptive field size measurements (Figures 2 & 3): Mysore et al (2010; Journal of Neuroscience). Paired electrophysiological recordings (Figures 3 & 4): Mahajan & Mysore (2022; Nature Communications), Schryver, Straka, and Mysore (2020; Journal of Neuroscience), Mysore & Knudsen (2014; Neuron), Mysore & Knudsen (2013; Nature Neuroscience), Asadollahi & Knudsen (2016; Nature Communications).
Data exclusions	For paired Imc-Imc recordings, site-pairs were included only when kynurenic acid iontophoresis caused significant reduction of responses at site B.
Replication	For our experimental data collection, we collected the data across 8 animals and across experiments (conducted on different days) to verify reproducibility of our findings. All attempts at replication were successful.
Randomization	Randomization was not relevant to this study because our study did not involve samples/organisms that were allocated into experimental groups.
Blinding	Blinding was not relevant to our study because there was no group allocation in our study.

Reporting for specific materials, systems and methods

We require information from authors about some types of materials, experimental systems and methods used in many studies. Here, indicate whether each material, system or method listed is relevant to your study. If you are not sure if a list item applies to your research, read the appropriate section before selecting a response.

Materials & experimental systems

n/a	Involved in the study	
☒	☐	Antibodies
☒	☐	Eukaryotic cell lines
☒	☐	Palaeontology and archaeology
☐	☒	Animals and other organisms
☒	☐	Clinical data
☒	☐	Dual use research of concern

Methods

n/a	Involved in the study	
☒	☐	ChIP-seq
☒	☐	Flow cytometry
☒	☐	MRI-based neuroimaging

Animals and other research organisms

Policy information about studies involving animals; ARRIVE guidelines recommended for reporting animal research, and Sex and Gender in Research

Laboratory animals	We performed experimental recordings in 8 head-fixed awake adult barn owls viewing a visual screen passively (Tyto alba). Both male and female birds were used; the birds were shared across studies. Ages of birds ranged between 1 to 4 years.
Wild animals	Study did not involve wild animals.
Reporting on sex	Results do not apply to only one sex, because birds were not selected specifically from any one sex. However, sex related details were not collected.
Field-collected samples	Study did not involve samples collected from the field.
Ethics oversight	All procedures for animal care and use were carried out following approval by the Johns Hopkins University Institutional Animal Care and Use Committee, and in accordance with NIH guidelines for the care and use of laboratory animals.

Note that full information on the approval of the study protocol must also be provided in the manuscript.
